# Approach to Thromboprophylaxis for Prevention of Venous Thromboembolism in COVID-19: Global Updates and Clinical Insights from India

**DOI:** 10.3390/clinpract12050080

**Published:** 2022-09-23

**Authors:** Nagarajan Ramakrishnan, Suresh Ramasubban, Ashit Hegde, Deepak Govil

**Affiliations:** 1 Department of Critical Care Medicine, Apollo Hospitals, Chennai 600006, Tamil Nadu, India; 2 Department of Critical Care, Apollo Gleneagles Hospital, Kolkata 700054, West Bengal, India; 3 Department of Critical Care and Medicine, PD Hinduja National Hospital and Medical Research Centre, Mumbai 400016, Maharashtra, India; 4 Institute of Critical Care and Anesthesiology, Medanta The Medicity, Gurgaon 122006, Haryana, India

**Keywords:** COVID-19, deep vein thrombosis, pulmonary embolism, thromboprophylaxis, venous thromboembolism

## Abstract

Venous thromboembolism (VTE) frequently occurs in patients with coronavirus disease-19 (COVID-19) and is associated with increased mortality. Several global guidelines recommended prophylactic-intensity anticoagulation rather than intermediate-intensity or therapeutic-intensity anticoagulation for patients with COVID-19-related acute or critical illness without suspected or confirmed VTE. Even though standard doses of thromboprophylaxis are received, many cases of thrombotic complications are reported; hence, appropriate and adequate thromboprophylaxis is critical for the prevention of VTE in COVID-19. In spite of an increased prevalence of VTE in Indian patients, sufficient data on patient characteristics, diagnosis, and therapeutic approach for VTE in COVID is lacking. In this article, we review the available global literature (search conducted up to 31 May 2021) and provide clinical insights into our approach towards managing VTE in patients with COVID-19. Furthermore, in this review, we summarize the incidence and risk factors for VTE with emphasis on the thromboprophylaxis approach in hospitalized patients and special populations with COVID-19 and assess clinical implications in the Indian context.

## 1. Introduction

Coronavirus disease-19 (COVID-19) has caused a huge social and economic breakdown worldwide. In India, as of 2 February 2022, there have been 41,630,885 confirmed cases of COVID-19 with 497,975 deaths [1]. Venous thromboembolism (VTE) is a significant global burden, but its incidence has remained relatively unchanged over the past decade until the COVID-19 outbreak [2]. In India, many cases of VTE are still undetected, and hence the incidence of VTE is underestimated [3].

VTE gained significant focus in the recent ongoing pandemic. Many cases may remain undiagnosed, and some deaths associated with COVID-19 appear to be thrombotic [4]. A recent meta-analysis has reported that the 21% of patients with COVID-19 developed VTE [5]. In a study by Lee AD et al., the incidence of VTE in India (pre-COVD era) was found to be 17.46 per 10,000 hospital admissions [6]. A systematic review and meta-analysis (n = 41,768) was conducted to compare the incidence of VTE in COVID-19 cohorts with non-COVID-19 cohorts. Cohorts from COVID-19 and non-COVID-19 did not differ significantly in VTE risk, except in the hospitalized intensive care unit (ICU) subgroups [7].

A higher mortality rate was observed in COVID-19 patients with VTE than in patients without VTE (23% [95% confidence interval, CI:14–32%] vs. 13% [95% CI: 6–22%]) [5], and a meta-analysis has revealed that the thromboembolism-related mortality risk increased by 74% [5]. Venous thrombosis in COVID-19 is associated with a mixture of macro- and micro-thrombosis. The patients with COVID-19 can exhibit (a) endotheliopathy, (b) hypercoagulability, and (c) coagulation activation [8]. In patients with severe COVID-19 infection and coagulopathy, appropriate thromboprophylaxis can improve the prognosis [9]. Several clinical practice guidelines, such as the American Society of Hematology (ASH) 2021, CHEST 2020, International Society of Thrombosis and Hemostasis (ISTH) 2020, and NICE 2021 have endorsed the use of pharmacological thromboprophylaxis for COVID-19 patients who are hospitalized unless contraindications exist [10,11,12,13,14,15]. In the absence of Indian data, our objective is to review the available global literature and provide clinical insights into our approach towards managing VTE in patients with COVID-19.

## 2. Methods

A comprehensive literature search was conducted on MEDLINE with PubMed interface (date of the last search: 31 May 2021) to include the relevant articles for this narrative review. Additional literature was also searched in Google scholar. The following search words were used in selecting the studies for this search: COVID-19, coronavirus, thromboembolism, thromboprophylaxis, VTE, anticoagulants, low molecular weight heparin, DOAC, India. These keywords were used in various combinations with the help of Boolean operators (e.g., ‘AND’, ‘OR’, ‘NOT’).

## 3. Incidence

A systematic review and meta-analysis by Malas MB et al. (2020) included 42 studies with 8271 SARS-CoV-2 patients. Overall, 21% (95% CI:17–26%) VTE rate was reported: ICU, 31% (95% CI: 23–39%). The incidence of deep vein thrombosis was 20% (95% CI: 13–28%): ICU, 28% (95% CI: 16–41%); postmortem, 35% (95% CI:15–57%). Overall, the pulmonary embolism rate was found to be 13% (95% CI: 11–16%): ICU, 19% (95% CI:14–25%); postmortem, 22% (95% CI:16–28%). The overall arterial thromboembolism rate was reported to be 2% (95% CI: 1–4%): in ICU, 5% (95%CI: 3–7%) [5].

According to the meta-analysis of 86 studies (33,970 patients), it was found that the incidence of VTE was 14.1%; 40.3% when screening was performed with ultrasound and 9.5% without screening [16]. In general, high prevalence rates of VTE are observed in hospitalized patients with COVID-19 [17]. Thrombotic events were reported to occur in 16.0% of hospitalized patients (ICU and non-ICU) with COVID-19; 7.1% were venous, and 11.5% were arterial [18]. The thrombotic complication rate was comparatively less in noncritically ill patients (4.7%) than in critically ill hospitalized patients with COVID-19 (18.1%) [18]. On the other hand, Hippensteel et al. found no significant differences between critically ill and non-critically ill patients in terms of mortality, though a higher prevalence of VTE was seen [19].

Despite anticoagulant thromboprophylaxis, a high prevalence of thrombotic complications (up to 34%) is reported in patients with COVID-19 in the ICU, and these rates were significantly higher in studies adopting systematic screening (duplex ultrasound of the limbs) (56.3%) than those relying on clinical suspicion (11.0%) [20]. Of these patients, deep vein thrombosis (DVT) occurred in 16.1% and pulmonary embolism (PE) in 12.6% and were associated with a high mortality rate [20]. A meta-analysis determining the association of COVID-19 with thromboembolic complications reported weighted mean prevalence of VTE as 31.3% (95% CI: 24.3–39.2%); DVT as 19.8% (95% CI: 10.5–34.0%), and PE as 18.9% (95% CI: 14.4–24.3%) [21].

The high prevalence of VTE in patients with COVID-19 has been observed in the elderly [22], obese [23], and patients with cardiovascular disease [24]. Overall, a wide variability is observed in VTE prevalence among multiple studies conducted worldwide regarding ethnicity, age, screening methods, and disease severity.

## 4. Pathophysiology

COVID-19 infection triggers VTE via several pathogenetic mechanisms, including endothelial dysfunction, systemic inflammation, and a pro-coagulatory state due to tissue factor pathway activation. The process of coagulation and normal clot formation and the fibrinolytic system are dysregulated in patients with COVID-19 infection [25]. Hyperinflammation subsequently results in severe instability of hemostasis classically observed in sepsis or disseminated intravascular coagulation (DIC). This may be characterized by a reduced thrombocyte count, prolonged prothrombin time (PT) and activated partial thromboplastin time (aPTT), decreased fibrinogen, and elevated levels of fibrinogen degradation products such as D-dimer [26,27]. These characteristic outcomes were also noticed in patients with COVID-19, demanding the need for intensive care and sometimes leading to death due to poor prognosis [27]. Another mechanism of COVID-19 immunothrombosis was proposed by Skendros P, et al. wherein the complement pathway is activated by SARS-CoV-2 infection, thereby enhancing C3 activation and generation of other complement pathway factors, including C3a and C5a. Further, C3a generation triggers platelet activation while C5a and platelet-derived thrombin induce thrombogenic neutrophil extracellular traps (NETs), which further leads to platelet activation and inflammation, which constitutes a complement/NET-driven vicious cycle of immunothrombosis [28]. Several mechanisms of hypercoagulability in sepsis, such as immune-based thrombotic mechanisms, complement, and macrophage activation, antiphospholipid antibody syndrome, elevated ferritin, and dysregulated renin-angiotensin system are presumed to be upregulated in COVID-19 [29].

**Figure 1:**
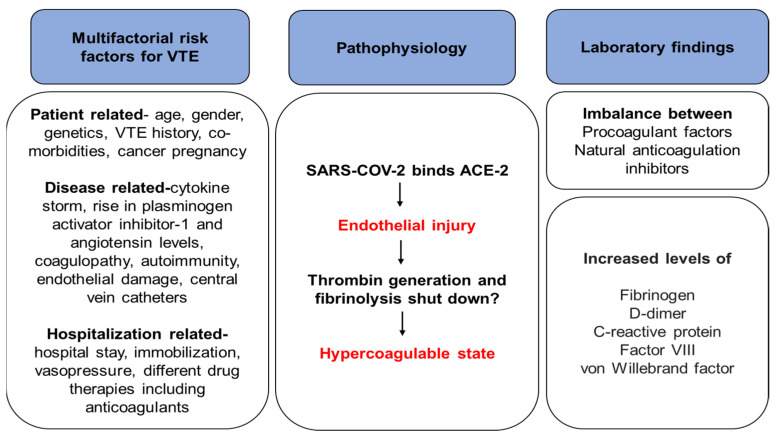
Associated risk factors and pathophysiology of VTE [33,34,35]. ACE-2: angiotensin-converting enzyme 2; SARS-CoV-2: severe acute respiratory syndrome coronavirus 2; VTE, venous thromboembolism.

Another proposed hypothesis is an imbalance between coagulation and inflammation processes leading to hypercoagulability that may be a cause of thrombosis in patients with COVID-19 [30]. Patients with COVID-19 show hyperactivated platelets leading to plasmatic cytokine load and thus have the potential to contribute to thrombo-inflammation [31]. Moreover, endothelial dysfunction is caused by lung inflammation, followed by hypoxemia and subsequent development of pulmonary microthrombi [32] (Figure 1).

## 5. Risk Factors Associated with VTE in COVID-19

Various risk factors specific to patients, diseases, and hospitals are associated with VTE in COVID-19 (Figure 1). Male gender, advanced age, obesity, critically ill ICU patients with severe COVID-19 infection, immobility, thrombogenic drugs (hormone replacement therapy, intravenous immune globulin, sildenafil, corticosteroids, erythropoietin, etc.) are associated with increased risk of VTE [36,37]. The previous history of VTE, cancer, sepsis, pregnancy, stroke, trauma, or surgery also can pose an additional risk for thrombosis [32]. Sedation, administration of vasopressor drugs, and use of central venous catheters are other ICU-related risk factors. Furthermore, ethnicity, cardiovascular disease, prior myocardial infarction, and high D-dimer levels are strongly associated with thrombotic events [17]. A retrospective study reported that hypertension (43.0%), diabetes (13.4%), and coronary heart disease (11.3%) were the predominant coexisting conditions observed in patients with COVID-19; of these, 48.0%, 14.0%, and 12.0%, respectively, belonged to the DVT group. Moreover, the number of ICU patients from the non-DVT group was significantly lower as compared to those from the DVT group (41.3% vs. 90%, *p* < 0.001) [2]. Notably, in patients with COVID-19, especially those who are critically ill, risk factors associated with thrombosis must be identified to determine which pharmacological prophylaxis is most appropriate.

## 6. Predictors of VTE in COVID-19

Clinical scoring systems such as Acute Physiology and Chronic Health Evaluation II score (APACHE II), Sequential Organ Failure Assessment (SOFA) score, and Padua prediction score (PPS), and biomarkers of coagulation such as D-dimer, fibrinogen, and platelet count predict the clinical course of the disease, need for hospitalization, in-hospital mortality, and patient outcomes [38]. The APACHE II score is an effective predictive model that determines in-hospital mortality and development of VTE and accounts for age and the presence of comorbidities. The APACHE II score is more efficacious in predicting mortality than the SOFA and confusion, urea, respiratory rate, blood pressure, and age 65 (CURB-65) scores [38]. Higher APACHE II, SOFA, and PPS scores are associated with poor prognosis and high mortality risk [38,39,40]. The Caprini risk score is another widely used and validated risk assessment model for predicting postoperative VTE in hospitalized patients. This score is based on summing individual risk factors and categorizes patients into very low (score 0), low (1 to 2), moderate (score 3 to 4), and high risk (score ≥ 5) categories [41,42,43,44].

The persistent rise in D-dimer level is a reasonable predictor of VTE and intensifies in severely ill patients infected with COVID-19. However, its cut-off level and prognostic values are still under investigation [45]. Interestingly, Dujardin et al. developed a prediction model for VTE in critically ill COVID-19 patients using C-reactive protein (CRP) and D-dimer biomarkers. D-dimer and elevated CRP are highly predictive of VTE in critically ill patients with COVID-19 (98%) [46]. Kampouri et al. demonstrated that the combination of Wells scores ≥ 2 and D-dimer ≥ 3000 ng/L is a good predictor of VTE [47] while Cohen et al. suggested that an increased Charlson Comorbidity Index, history of cardiovascular disease, and ICU level of care are predictors of VTE along with D-dimer at or above four times the upper limit of normal [48]. These predictors of VTE can be useful to guide empiric thromboprophylaxis in the absence of diagnostic imaging. Box 1 describes the importance of D-dimer levels in the prediction of VTE during COVID-19.

Box 1. **D-dimer levels [49,50,51].**

○ Elevated D-dimer levels are independently associated with thrombotic events, which could indicate an early coagulopathy and severity of COVID-19.
○ Elevation of D-dimer levels precedes multiorgan failure and overt DIC resulting in increased mortality.
○ High mortality rates are associated with elevated D-dimer levels at admission and marked increases in D-dimer levels over time (three- to four-fold).
○ A patient with COVID-19 should not be admitted or undergo imaging for DVT/PE if D-dimer levels are elevated alone without any additional symptoms or signs of VTE.
○ Rapidly increased D-dimer with clinical deterioration should raise suspicion of PE.

Abbreviations: COVID-19, coronavirus disease 2019; DIC, disseminated intravascular coagulation; DVT, deep vein thrombosis; PE, pulmonary embolism; VTE, venous thromboembolism.

## 7. Thromboprophylaxis

Though several studies evaluated the different molecules for prophylactic and therapeutic usage in VTE, it is an evolving area and ideal options are being further explored (Box 2). The points to be considered during the selection of VTE thromboprophylaxis include risk of major bleeding, comorbidities, patient preference, compliance, underlying comorbidities, accessibility, and cost [52]. Pneumonia and admission to the hospital or ICU are the key factors that qualify patients for thromboprophylaxis [53]. Thromboprophylaxis with low-molecular-weight heparin (LMWH) during hospitalization reduces VTE events in non-COVID-19 medically ill patients [54]. It is recommended by the American College of Chest Physicians Evidence-Based Clinical Practice Guidelines (9th ed) [55]. CHEST 2020 guidelines recommend anticoagulant thromboprophylaxis with LMWH or fondaparinux over unfractionated heparin (UFH) in acutely ill hospitalized patients with COVID-19. Further, instead of DOAC, it was suggested to use LMHW, fondaparinux or UFH. In contrast, anticoagulant thromboprophylaxis with LMWH over UFH and LMWH or UFH over fondaparinux or a DOAC were recommended in critically ill patients hospitalized with COVID-19. In both patient populations (acutely ill and critically ill hospitalized), the dose of anticoagulant thromboprophylaxis should be the current standard-dose over intermediate (LMWH BID or increased weight-based dosing) [14]. However, there is insufficient data regarding bleeding risk in COVID-19 patients and, hence, caution is to be exercised while increasing the dosages beyond the guideline-recommended doses. On the other hand, the ASH 2021 guidelines suggested no preferred choice of anticoagulant thrombophylactic agent and suggested the use of a treatment agent based on availability, resource settings, familiarity, and the aim of minimizing exposure to COVID-19-infected patients as well as patient-specific factors (such as kidney function, history of heparin-induced thrombocytopenia, gastrointestinal tract absorption difficulties) [13].

Data support the role of thromboprophylaxis in reducing VTE events and mortality associated with COVID-19 [56,57]. A Dutch study demonstrated a reduced risk for overall mortality in the second wave of the COVID-19 pandemic, but the rate of thrombotic complications remained high, equivalent to the first wave [58].

For COVID-19 patients without suspected or confirmed VTE, the American Society of Hematology 2021 recommended prophylactic anticoagulation over intermediate or therapeutic-intensity anticoagulation [12]. Contraindication in renal and hepatic impairment [59] and the possibility of several drug–drug interactions, particularly with the antiviral agents, need to be considered while initiating a pharmacological thromboprophylaxis [60,61,62].

A global survey of 515 clinicians from 41 countries evaluated current practice and experience in managing COVID-19-associated coagulopathy, most clinicians (78%) suggested thromboprophylaxis for all the people from the in-patient department, with the majority preferring the use of LMWH or UFH. In this survey, the majority of respondents (43%) reported that patients on therapeutic anticoagulation had a higher bleeding risk [63]. However, studies have reported a cumulative incidence of VTE despite receiving prophylactic or therapeutic anticoagulation [64,65], thus, suggesting the optimization of the management strategy of VTE in COVID-19.

Box 2. **Different thromboprophylaxis regimens.**

○ LMWH is preferred due to lesser exposure to health workers and partial thromboplastin time monitoring is not required [59].
○ UFH is less preferred due to drawbacks such as increased staff exposure, need for laboratory monitoring, and occurrence of thrombocytopenia [59].
○ Mechanical thromboprophylaxis is used when pharmacological thromboprophylaxis is contraindicated. However, its additional use is not recommended in critically ill patients receiving pharmacological prophylaxis [59].
○ For hospitalized patients:
  - Acutely ill/critically ill: With the absence of contraindication, anticoagulant (AC) thromboprophylaxis should be used over no AC prophylaxis [14].
  - Acutely ill: AC prophylaxis with LMWH or fondaparinux over UFH; LMWH/fondaparinux/UFH should be used over DOACs [14].
  - Critically ill: AC prophylaxis with LMWH over UFH; LMWH/UFH is recommended over fondaparinux/DOACs [14,66].
  - All patients: In-patient thromboprophylaxis is preferred over in-patient plus extended thromboprophylaxis [14].

Abbreviations: AC, anticoagulant, DOACs, direct oral anticoagulants; LMWH, Low-molecular-weight heparins; UFH, unfractionated heparin.

## 8. Standard vs. Intermediate vs. Therapeutic Regimens or Intensity

Clinical trials such as the INSPIRATION trial (the intermediate versus standard-dose prophylactic anticoagulation in critically ill patients with COVID-19), and the other international multiplatform RCT, including randomized, embedded, multifactorial adaptive platform trial for community-acquired pneumonia (REMAP-CAP), accelerating COVID-19 therapeutic interventions and vaccines-4 antithrombotics in-patient platform trial (ACTIV-4a), antithrombotic therapy to ameliorate complications of COVID-19 (ATTACC), HEP-COVID and medically ill hospitalized patients for COVID-19 thrombosis extended prophylaxis with rivaroxaban therapy (MICHELLE trial) were conducted to determine the optimal anticoagulant dosing regimen for thromboprophylaxis [67,68,69,70]. Multiple ongoing trials assessing the role of antithrombotic agents in critically ill patients with COVID-19 encompassing ICU and non-ICU patients.

The findings from the INSPIRATION trial showed comparable results in patients with COVID-19, treated with intermediate (1 mg/kg daily) and standard-dose (40 mg daily) prophylactic anticoagulation with enoxaparin for venous or arterial thrombosis, treatment with extracorporeal membrane oxygenation, or mortality within 30 days [67]. The potential reason for this may be insufficient intermediate doses to prevent thrombotic events. Although bleeding events were rare, an intermediate dose of enoxaparin showed nonsignificant higher bleeding events (2.5%; *p* = 0.33) and significant events of severe thrombocytopenia (2.2%; *p* = 0.01) [67]. Nevertheless, a study by Albani F et al. showed that enoxaparin lowered hospital stays, ICU admission, and death rate supporting its use in thromboprophylaxis of patients with COVID-19 [71]. The multiplatform RCTs (that involved an unprecedented collaboration between trial investigators, platforms, and countries), REMAP-CAP, ATTACC, and ACTIV4a showed that in patients with severe COVID-19, therapeutic anticoagulation compared to usual care pharmacological thromboprophylaxis did not improve hospital survival (62.7% vs. 64.5%) or days free of organ support (1 day (IQR–1, 16) vs. 4 days (IQR–1, 16) and, the risk of major bleeding was found to be 3.8% and 2.3% in patients receiving therapeutic anticoagulation and pharmacological thromboprophylaxis, respectively [68]. There is evidence that the therapeutic dose is superior to usual-care venous thromboprophylaxis in patients with moderate COVID-19 about organ support-free days in every D-dimer subgroup as well as overall improvement in morbidity and mortality [72]. Moreover, the major bleeding rate was < 2% among patients receiving therapeutic anticoagulation.

Some patients with COVID-19 experience recurrent clotting of access devices or extracorporeal circuits, despite prophylactic anticoagulation. In such patients, clinicians may increase the anticoagulation strength gradually or substitute it with other anticoagulants. Clinical judgment and individual patients’ bleeding risk should be considered while switching or increasing the anticoagulation strength [73].

In the ASH 2021 guidelines, prophylactic anticoagulation over intermediate or therapeutic anticoagulation is recommended for patients who have COVID-19-associated illness but no signs or symptoms of VTE [13]. ISTH recommends intermediate prophylactic doses both in moderately and severely ill COVID-19 patients [74]. The American College of Chest Physicians (ACCP) prefers standard prophylaxis doses in both patient categories [14].

## 9. Duration of Thromboprophylaxis; in-Hospital vs. Extended

Pre-COVID-19 RCTs such as the MARINER [75] and MAGELLAN [76] studies revealed that extended thromboprophylaxis (around 30–45 days) does not provide a clear net benefit in patients with medical illness. The pre-COVID-19 guidelines also advised against the usage of extended prophylaxis (i.e., 30 to 42 days) beyond hospital discharge [14,77]. CHEST 2020 guidelines for COVID-19 suggest in-patient thromboprophylaxis alone is preferable over in-patient plus extended thromboprophylaxis after discharge from the hospital [14]. Despite this, patients with COVID-19 with low bleeding risk should be considered for extended thromboprophylaxis if new data on post-discharge VTE and bleeding show a net benefit of such a measure [14].

Data on VTE occurrence and the need for thromboprophylaxis post-hospitalization with COVID-19 is limited. Two observational studies (Salisbury et al. 2020 and Roberts et al. 2020) from the United Kingdom showed an incidence rate of VTE to be 2.6% and 4.8 per 1000 discharges at 42 days showing no increase in the risk of VTE compared with hospitalization with other acute medical illness [78,79]. Another multicenter prospective registry study by Giannis et al., including 4906 hospitalized patients with COVID-19, reported a high occurrence of thrombotic events after hospital discharge and associated death that was reduced significantly with post-discharge anticoagulation. Most importantly, thromboprophylaxis after hospital discharge was administered to 13.2% of patients, mostly at prophylactic dosages, and this lowered the risk of major thromboembolic events and death by 46% [80].

**Figure 2:**
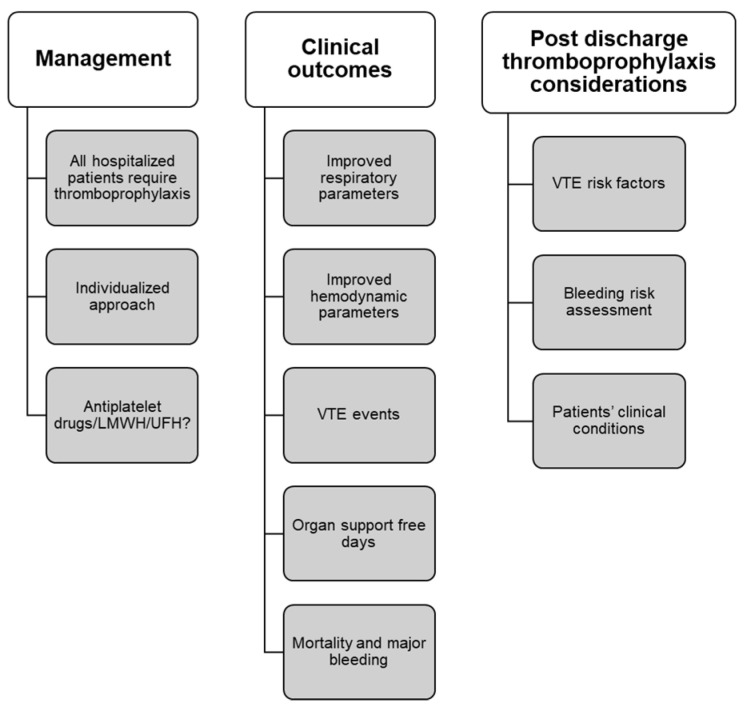
Management of VTE in-hospital and post-discharge [33,81]. LMWH, Low-molecular-weight heparins; UFH, unfractionated heparin; VTE, venous thromboembolism.

Guidelines, including the Scientific and Standardization Committee (SSC) of the ISTH (clinical guidance on the diagnosis, prevention, and treatment of VTE in hospitalized patients with COVID-19 [2020]) and the American College of Chest Physicians Guideline and Expert Panel Report (prevention, diagnosis, and treatment of VTE in patients with COVID-19 [2020]), for extended post-discharge thromboprophylaxis in patients with COVID-19 are also conflicting, with either no thromboprophylaxis or a customized approach as per the patient’s thrombotic and bleeding risk factors [14,74]. ISTH recommends extended post-discharge thromboprophylaxis for all COVID-19 patients with a high risk of VTE criteria and can be continued for 14 days to 30 days [74]. Furthermore, the ACCP panel suggested that “extended thromboprophylaxis would result in net benefit in patients with COVID-19 at low bleeding risk if the risk of symptomatic VTE would be above 1.8% at 35 to 42 days after hospital discharge [14].” The need for post-discharge thromboprophylaxis is being investigated in clinical trials. Considering individual patient VTE risk factors is important; the risk of VTE is likely to outweigh the risk of bleeding, and the patients should be educated about VTE symptoms during discharge (Figure 2). Box 3 summarizes the dose and duration of thromboprophylaxis.

Box 3. **Dose and duration of thromboprophylaxis.**

○ A standard dose of LMWH should be considered in all hospitalized patients after assessing bleeding risk [63].
○ Prophylactic doses of anticoagulation are preferred over therapeutic doses in patients with COVID-19 who do not have suspected or confirmed VTE [13].
○ In the case of presumed VTE, the dose may be escalated from a prophylactic to a therapeutic or an intermediate dose [11].
○ Extended thromboprophylaxis is not recommended routinely and should only be considered if VTE risk continues to be high during discharge with low bleeding risk [14].

Abbreviations: COVID-19, coronavirus disease-2019; LMWH, low-molecular-weight heparins; VTE, venous thromboembolism.

## 10. Considerations in Special Population

### 10.1. Renal Impairment

According to the American College of Chest Physicians Guideline and Expert Panel Report (prevention, diagnosis, and treatment of VTE in patients with COVID-19 [2020]), patients with severe renal impairment (creatinine clearance < 30 mL/min) who are at high bleeding risk and receiving LMWH or fondaparinux need to undergo dosage adjustment or should be switched to UFH. All such patients with mild-to-moderate renal failure should be closely monitored for bleeding risk [14]. Enoxaparin is the most studied LMWH in patients with renal dysfunction. Dose adjustment of enoxaparin is not required in mild-to-moderate renal impairment, while it can be used with dose adjustment in patients with severe renal impairment [82,83].

### 10.2. Cancer

Patients with cancer are highly vulnerable to COVID-19 infection, and VTE is common in both cancer and COVID-19. Patients with active cancer and COVID-19 can receive pharmacological VTE prophylaxis unless contraindicated [84]. However, due to limited evidence and a high risk of bleeding and thrombosis, a vigilant benefit–risk assessment is necessary when deciding the treatment for these patients. In addition, it is imperative to consider the assessment of the dose and duration of the anticoagulation regimen to secure better clinical outcomes in these patients [85]. A detailed consideration of the duration of prophylaxis and delay of chemotherapy (often 14–28 days) post-COVID-19 is also vital in cancer patients. A study (Paredes-Ruiz et al., 2021) reported that a standard thromboprophylaxis dose of LMWH can prevent VTE in patients with non-hematological cancer hospitalized because of COVID-19 [86].

### 10.3. Overweight or Obese

The Scientific and Standardization Committee of the ISTH recommends that hospitalized patients with COVID-19 and obesity (body mass index, BMI >30 kg/m^2^) or morbid obesity (BMI > 40 kg/m^2^) undergo weight-based dosing of LMWH for thromboprophylaxis [74]. High-dose thromboprophylaxis (up to 50%) should be considered in obese patients (actual body weight or BMI) [74].

### 10.4. Pregnancy

As per the Royal College of Obstetricians and Gynecologists (RCOG), all pregnant women with confirmed or suspected COVID-19 should be given prophylactic LMWH (for 10 days post-discharge) unless childbirth is expected within 12 h. A similar management strategy is recommended for women admitted with confirmed or suspected COVID-19 within 6 weeks after delivery, except for the difference in thromboprophylaxis duration (it should continue for their days of hospitalization) [87]. Until validated clinical trial data is available, prophylactic doses of anticoagulants could be advised by clinicians based on disease severity and thrombotic risk. Generalizing the anticoagulant strategy to these special populations with COVID-19 is challenging, and emerging data will aid in deciding the appropriate doses for thromboprophylaxis in these patients.

Based on the available evidence about thromboprophylaxis use in non-COVID-19 patients, considerations for thromboprophylaxis in special populations are summarized in Box 4.

Box 4. **Considerations for thromboprophylaxis in special population.**

○ The available data suggest that LMWH can accumulate in patients with a creatinine clearance < 30 mL/min with therapeutic doses. This can increase the patient’s risk of bleeding by two-fold as compared to the patients with normal creatinine clearance [88].
○ Evidence suggests the requirement of dose reduction for enoxaparin, whereas tinzaparin can be used without dose reduction [89,90,91].
○ High-risk out-patients with cancer with a Khorana score of two or higher before initiating new systemic chemotherapy may be offered thromboprophylaxis with apixaban, rivaroxaban, or LMWH [92].
○ Hospitalized cancer patients with creatinine clearance of ≥ 30 mL/min, LMWH or fondaparinux or UFH. DOACs are not recommended routinely [93].
○ Thromboprophylaxis with a weight-adjusted dose of LMWH is preferred in patients with obesity [74].

Abbreviations: DOACs, direct oral anticoagulants; LMWH, low-molecular-weight heparins; UFH, unfractionated heparin.

## 11. Clinical Implications of VTE in COVID-19 in Indian Scenario

Indian data about thrombotic complications in patients with COVID-19 are scarce, which may be due to non-specific symptoms and inadequate access to imaging techniques in resource-limited settings. The present recommendations are limited in terms of guidance on detailed evaluation and focus more on primary disease management rather than preempting thrombosis in COVID-19 patients. Retrospective registries of Indian patients demonstrate that VTE is not a rare condition in India, but the knowledge and awareness among the health care practitioners is minimal [94,95,96].

A prospective observational study from India showed that thromboprophylaxis played a substantial role in preventing DVT in non-COVID-19 patients [95]. Risk stratification for managing VTE in all patients with COVID-19 is important. Asian venous thromboembolism guidelines were introduced in 2012 and updated in 2016 to answer the VTE-related questions of clinicians from Asia and India [96,97]. Recently, the clinical guidance from the Ministry of Health and Family Welfare of the Government of India has recommended anticoagulation strategy in moderate cases provided there is an absence of contraindication or bleeding risk [98]. The Indian Society of Critical Care Medicine (ISCCM) (2020) updated position statement recommends considering all COVID-19 hospitalized patients as having a high risk of VTE and using a standard prophylactic dose of UFH or LMWH [99]. Management of VTE can be enhanced in India with continuing medical education initiatives, the institution of hospital-based guidelines, a multidisciplinary approach, routine clinical investigation, and reporting of VTE-related issues. For patient populations infected with the Omicron variant, it is important to note that in the absence of published data on the Omicron variant, the management of VTE may remain the same as described in this review (based on the international guidelines) and it will purely depend on the severity of the disease and patient profile. 

## 12. Limitations and Future Directions

Although this review presents comprehensive data on managing VTE in COVID-19 patients, it is important to note that it presents one-year-old data due to a predefined time limit set for the literature search. In addition, most of the studies included were performed during the initial waves of COVID-19, and SARS-CoV-2 is evolving continuously; therefore, readers need to be cautious while interpreting the data. Furthermore, the lack of real-world studies and randomized clinical trials that involve a large sample size and place particular emphasis on special populations such as pregnant women and the elderly has limited the strength of this review in terms of discerning the role of thromboprophylaxis in VTE management during the COVID-19 pandemic. Therefore, randomized clinical trials and real-world evidence studies are needed to understand the impact of thromboprophylaxis on mortality and morbidity with different regimens’ (LMWH, DOAC, LMWH + DOAC, antiplatelets, other antithrombotics) doses, and duration of the regimen based on COVID-19 severity. Special populations, including elderly and pregnant women, should be considered while designing these studies. Analyzing and comparing the results of multiplatform trials should consider the technical and interpretational complexities while applying the results to clinical practice. Ongoing/registered clinical trials evaluating anticoagulant therapy in patients with COVID-19 are summarized in Table S1 [100,101,102,103,104,105,106,107,108,109,110,111,112,113,114,115,116,117,118,119,120].

## 13. Conclusions

VTE frequently occurs in patients with COVID-19 and is associated with increased mortality. Appropriate and adequate thromboprophylaxis is critical for preventing VTE in all hospitalized patients with COVID-19. Validated data from clinical trials and real-world studies evaluating different prophylactic strategies for VTE during COVID-19 is awaited.

## Data Availability

Not applicable.

## Supplementary Materials

The following supporting information can be downloaded at: https://www.mdpi.com/article/10.3390/clinpract12050080/s1, Table S1: Ongoing/registered clinical trials evaluating anticoagulant therapy in patients with COVID-19 [100,101,102,103,104,105,106,107,108,109,110,111,112,113,114,115,116,117,118,119,120].
