# Supplementary material for: Approach to Thromboprophylaxis for Prevention of Venous Thromboembolism in COVID-19: Global Updates and Clinical Insights from India
Source: Clin Pract. 2022 Sep 23;12(5):766–81. doi: 10.3390/clinpract12050080 (PMC9601217; doi:10.3390/clinpract12050080)
Supplement: Supplementary file 1 [file clinpract-12-00080-s001.zip › clinpract-1851393-supplementary.pdf]

Supplementary Material

Table S1. Ongoing/registered clinical trials evaluating anticoagulant therapy in patients with COVID-19.

Identifier	Study design	Intervention	Population	Primary Endpoints
NCT04730856 [1]<br>**Spain**	Open label<br>N=600	Tinzaparin (SC):<br>Follow-up phase 1 (Day 15 to day 30)<br>Follow-up phase 2 (At 6 months)	• Adult patients (≥18 years) with 50-100 kg weight<br>• Signed informed consent<br>• Presence of at least, one of the following risk criteria:<br>○ SpO_2:<94%<br>○ P/F <300 mmHg<br>○ DD >1000 μg/L<br>○ PCR >150 mg/L<br>○ IL-6 >40 pg/mL	• Reduction of suspicion of systemic thrombotic symptomatic events<br>• Need for MV<br>• Progression of WHO Scale<br>• Overall survival<br>• Length of hospital and ICU stay<br>(during 30 days)
NCT04623177 [2]<br>**Spain**	Cohort<br>N=950	Anticoagulant dose (≥150 IU/kg/24 h) of LMWH within the first 48 h after the ICU admission	• All COVID-19 patients admitted during 12 March to 01 September 2020	• ICU mortality from admission to ICU discharge (during 1 month)
NCT04616846 [3]<br>**France**	Open Label<br>N=120	-	• Adult patients (≥18 years)<br>• Patient treated for cancer<br>• Patient being screened for VTE at least one time 7 weeks after the COVID-19 diagnosis at two time point (day 1-10 after COVID-19 testing, and day 20-25 after COVID-19 testing).	• Rate of VTE<br>• DVT and/or PE<br>(From day 9 to Day 42)
NCT04542408 [4]<br>**Germany**	Double-blind<br>RCT<br>N=172	Anticoagulation Agents<br>(Edoxaban and/or high dose LMWH)<br>Low dose LMWH or Placebo	• Adult patients (≥18 years)<br>• Hospitalization on normal ward or ICU	• All-cause mortality and/ or VTE and/ or ATE during follow-up (42 days).

Identifier	Study design	Intervention	Population	Primary Endpoints
			• Diagnosis of COVID-19 (within 10 days) and troponin ≥ULN and/or D-dimer ≥0.5 mg/L	
NCT04528888 [5]<br>**Italy**	Open label<br>N=210	• Enoxaparin (SC) at 4000 UI OD, increased to 6000 UI OD for patients weighting >90 kg was given up to ICU discharge.<br>• After ICU discharge it may be continued or interrupted.	• Need for either NIV or invasive ventilation from >24 h<br>• MV from <96 h<br>• P/F ratio <150<br>• D-dimer level >6 ULN<br>• PCR >6-fold ULN	• All-cause mortality at day 28
NCT04512079 [6]<br>**United States**	Open label<br>N=3600	• Apixaban: 5 mg Q12h; 2.5 mg Q12h for patients with at least two of three of age ≥80 years, weight ≤60 kg or serum creatinine ≥1.5 mg/dL.	• Fever >38°C<br>• SpO_2 ≤94<br>• Abnormal laboratory marker (at least 1):<br>D-dimer ≥1.0 μg/mL CRP >2 mg/L, Ferritin >300 μg/L, Lymphopenia <1500 cells/m^3	• All-cause mortality<br>• Need for MV<br>• Systemic thromboembolism<br>• Need for surgical intervention<br>• Ischemic stroke<br>(during 30 days)
NCT04505774 [7]<br>**United States**	Multicenter RCT<br>N=2000	• Heparin	• Adult patients (≥18 years)<br>• Enrolled within 72 h of hospital admittance or 72 h of positive COVID test<br>• Expected to require hospitalization for >72 h	• Organ support free days<br>(during 21 days).

Identifier	Study design	Intervention	Population	Primary Endpoints
NCT04492254 [8]<br>**United Kingdom**	Open label, multicenter, multi-national RCT<br>N=1370	• Single-dose SC injection of enoxaparin sodium 40 mg/0.4 mL.	• Signed informed consent<br>• Men or women, age ≥55 years<br>• Presence of at least, one of the following risk criteria:<br>○ Age ≥70 years, BMI >25 kg/m^2, COPD, DM, CVD, Corticosteroid use	• ARDS, Admission ICU, MV, intubation requirement, CPAP/NIV, ECMO (during 21 days)
NCT04486508 [9]<br>**Iran**	Multicenter open-label 2x2 factorial RCT<br>N=600	• Intermediate dose Enoxaparin/ unfractionated heparin according to CLCR and weight<br>• Atorvastatin: 20 mg	• Adult patients (≥18 years) admitted to ICU within 7 days of initial hospitalization, who do not have firm indication mechanical valve, AF, VTE, or left ventricle thrombus.<br>• Informed consent.<br>• Estimated survival of at least 24 h	• Acute VTE, arterial thrombosis, treatment with ECMO, or all-cause mortality (30 days).
NCT04420299 [10]<br>**Spain**	Single-blind RCT<br>N=120	• Bemiparin at therapeutic dose for 10 days	• Informed consent.<br>• 18 Years and older<br>• D-dimer >500 ng/mL<br>• Patient admitted to hospital	• Death.<br>• ICU admission.<br>• Need for either NIV or MV<br>• Progression to moderate/severe respiratory distress syndrome, VTE, acute MI or stroke. (During 10±1 days)
NCT04416048 [11]<br>**Germany**	Open Label<br>N=400	• Rivaroxaban: 20 mg (15 mg for subjects with an eGFR ≥30 mL/min/1.73m^2 and <50 mL/min/1.73m2) OD for 7 days.	• Informed consent<br>• Patients of either sex with ≥18 years of age, with diagnosis moderate to severe COVID-19 with D-Dimer >1.5 ULN<br>• Cardiac injury reflected with cTnT >2.0 ULN	• DVT and/or fatal or non-fatal PE, arterial thromboembolism, MI, non-hemorrhagic stroke, all-cause mortality or need for MV (during 35 days).

Identifier	Study design	Intervention	Population	Primary Endpoints
		• In case of hospitalization for >7 days: rivaroxaban was continued until discharge.		
NCT04409834 [12]<br>**United States**	Open label<br>N=750	• Unfractionated Heparin IV: targeting an aPTT of 1.5-2.5 times the control<br>• Enoxaparin 1 mg/kg SC Q12h<br>• Clopidogrel 300 mg PO x1, then clopidogrel 75 mg PO QD	• Adult patients (men or women)<br>• Acute infection with SARS-CoV2<br>• Currently admitted to an ICU	• VTE or arterial thrombotic events, Hierarchical composite: mortality, PE, clinically evident DVT, type 1 MI, ischemic stroke, systemic embolism or acute limb ischemia (during 28 days)
NCT04408235 [13]<br>**Italy**	multicenter, open label, investigator sponsored, two arms study RCT<br>N=300	Enoxaparin<br>Low-Dose LMWH: enoxaparin 4000 IU daily; High dose LMWH: 70 IU/kg BID.	• Severe pneumonia:<br>o Respiratory Rate ≥25 breaths /min<br>o Arterial oxygen saturation ≤93% at rest on ambient air<br>o PaO2/FiO2 ≤300 mmHg<br>• Coagulopathy:<br>o D-dimer >4 ULN<br>o Sepsis-Induced Coagulopathy (SIC) score >4<br>o No need for MV	• Death<br>• AMI<br>• ATE or VTE<br>• Need for either NIV, CPAP, or invasive MV
NCT04406389 [14]<br>**United States**	Open label<br>N=186	• CLCR ≥30 mL/min: Enoxaparin sodium 0.5 mg/kg SC every 12 h	• Adult patients (≥18 years)	• Mortality (during 30 days)

Identifier	Study design	Intervention	Population	Primary Endpoints
		• CLCR <30 mL/min: Enoxaparin sodium 0.5 mg/kg SC every 24 h<br>• Unfractionated heparin: 7,500 U SC every 8 h<br>• Fondapariniux: 2.5 mg SC daily<br>o Dose by weight:<br>≥100 kg: 10 mg daily<br>≥50 to <100 kg: 7.5 mg daily<br><50 kg: 5 mg daily	• Need for ICU admission, NIV, MV, BiPAP, 100% non-rebreather mask, or HFO of at least 4 L/min nasal cannula.<br>• D-dimer level: >700 ng/mL	
NCT04401293 [15]<br>**United States**	Open-label multicenter RCT<br>N=308	Enoxaparin:<br>Full dose LMWH anticoagulation therapy	• Written informed consent within 72 h of hospital admission or transfer from another facility within 72 h of index presentation.<br>• Men or non-pregnant women adult, aged ≥18 years.<br>• D-Dimer: >4.0 times ULN,<br>• SIC score of ≥4	• ATE, VTE, and all-cause mortality at day 30 ± 2 days.<br>• Risk of MI, stroke, SE, VTE, DVT, asymptomatic proximal DVT, non-fatal PE, and all-cause mortality (during 30 ± 2 days).
NCT04367831 [16]<br>**United States**	Open Label<br>N=100	• Heparin Infusion:<br>Unfractionated heparin infusion at 10 units/kg/hour with goal anti-Xa 0.1 - 0.3 U/mL.<br>• Enoxaparin/Lovenox Intermediate Dose:<br>If estimated GFR ≥30 mL/min: enoxaparin 1 mg/kg SC daily.	• Transfer from nonparticipating to participating ICU<br>• Patients not on therapeutic anticoagulation and who were already admitted to participating ICU within 5 days of trial initiation	• VTE or ATE events in ICU (during 30 days)
NCT04373707 [17]<br>**France**	Single<br>N=602	• Enoxaparin: 4000 IU for OD in patients admitted in medical ward	• Adult patient hospitalized with confirmed COVID-19 infection	• DVT, PE, VTE related death.

Identifier	Study design	Intervention	Population	Primary Endpoints
		4000 IU for BID in patients admitted in the ICU.<br>• In patients with severe renal insufficiency (GFR=15-30 mL/min/1.73m²), LMWH doses will be reduced by 50%.	• Signed informed consent<br>• Patient affiliated to the Social Security	
NCT04360824 [18]<br>**United States**	Multicenter, randomized, open-label study<br>N=170	• Prophylactic dose enoxaparin<br>o BMI <30 kg/m²: 40 mg SC daily<br>o BMI ≥30 kg/m²: 30 mg or 40 mg SC BID.	• Age ≥18 years<br>• Requires hospital admission<br>• Modified ISTH Overt DIC score ≥ 3	• Risk of all-cause mortality (30 days post-intervention)
NCT04345848 [19]<br>**Switzerland**	Multicentric, open label RCT<br>N=200	• Two different doses of anticoagulation	• Adult patient admitted to non-critical with D-dimer levels >1000 ng/mL at admission<br>• Or admitted to an ICU.	• Risk of ATE or VTE, DIC, and all-cause mortality (During 30 days)
NCT04662684 [20]<br>**Brazil**	Randomized, open-label study<br>N=320	• Drug: Rivaroxaban 10 mg	• Male and nonpregnant female patients 18 years of age or older<br>• Positive RT-PCR assay for SARS-CoV-2 in a respiratory tract sample<br>• Pneumonia confirmed by chest imaging<br>• Additional risk factors for VTE, as indicated by a total modified International Medical Prevention Registry on VTE (IMPROVE) risk score of 4 or higher	• VTE and VTE related-death [Time Frame: at day 35 +/- post hospital discharge]

Identifier	Study design	Intervention	Population	Primary Endpoints
			• Have received thromboprophylaxis with low-molecular-weight heparin, fondaparinux, or unfractionated heparin during the index hospitalization	
NCT04508023 [21]<br>**United States**	Multicenter, randomized, placebo-controlled, pragmatic phase 3 study<br>N=4000	• Drug: Rivaroxaban<br>• Other: Placebo<br>• Other: Standard of Care (SOC)	• Age ≥18 years<br>• Diagnosed with COVID-19<br>• Symptoms attributable to COVID-19 (example, fever, cough, loss of taste or smell, muscle aches, shortness of breath, fatigue)<br>• Initial treatment plan does not include hospitalization<br>• Presence of at least 1 additional risk factor: a) age ≥ 60 years; prior history of VTE; thrombophilia; coronary artery disease (CAD); peripheral artery disease (PAD); cerebrovascular disease or ischemic stroke; cancer (other than basal cell carcinoma); diabetes requiring medication; heart failure; BMI ≥ 1.	• Time to first occurrence of a composite endpoint of symptomatic vte, mi, ischemic stroke, acute limb ischemia, non-cns systemic embolization, all-cause hospitalization and all-cause mortality
NCT04409834 [12]<br>**United States**	Multicenter, open-label, randomized, study<br>N=750	• Drug: Unfractionated Heparin IV<br>• Drug: Enoxaparin 1 mg/kg<br>• Drug: Clopidogrel	• Age ≥18 years (male or female)<br>• Acute infection with severe acute respiratory syndrome coronavirus 2 (SARS-CoV2)<br>• Currently admitted to an ICU	• VTE or ATE [Time Frame: 28 days or until hospital discharge, whichever earlier.

Identifier	Study design	Intervention	Population	Primary Endpoints
		• Drug: Unfractionated heparin SC<br>• Drug: Enoxaparin 40 mg/0.4 mL Injectable Solution		

AF, high-risk atrial fibrillation; aPTT, activated partial thromboplastin time; ARDS, Acute Respiratory distress syndrome; AMI, Acute Myocardial Infarction; ATE, arterial thromboembolism; BID, two times daily; BMI, body mass index; CLCR, creatinine clearance; CPAP, Continuous positive airway pressure; COPD, Chronic obstructive pulmonary disease; CRP, C-reactive protein; CVD, Cardiovascular disease; DD, Droplet digital; DIC, disseminated intravascular coagulation; DM, diabetes mellitus; DVT, Deep vein thrombosis; ECMO, Extracorporeal membrane oxygenation; GFR, glomerular filtration rate; HFO, high flow oxygen; ICU, intensive care unit; IL-6; interleukin-6; ISTH, International Society on Thrombosis and Haemostasis; LMWH, Low Molecular Weight Heparin; MI, myocardial infarction; MV, mechanical ventilation; PCR, polymerase chain reaction; PE, pulmonary embolism; P/F, pAO2/FiO2; RCT, randomized controlled trial; ULN, upper limit of normal; VTE, VTE.
The last Web search was done on 02 June 2021.

References

1. Clinical Trial NCT04730856. Available from: https://clinicaltrials.gov/ct2/show/NCT04730856?term=prophylactic+anticoagulants+for+thromboembolism+in+covid-19&draw=4&rank=20#contacts. Accessed on 2nd June 2021.
2. Clinical Trial NCT04623177. Available from: https://clinicaltrials.gov/ct2/show/NCT04623177?term=prophylactic+anticoagulants+for+thromboembolism+in+covid-19&draw=4&rank=17. Accessed on 2nd June 2021.
3. Clinical Trial NCT04616846. Available from: https://clinicaltrials.gov/ct2/show/NCT04616846?term=prophylactic+anticoagulants+for+thromboembolism+in+covid-19&draw=2. Accessed on 2nd June 2021.
4. Clinical Trial NCT04542408. Available from: https://clinicaltrials.gov/ct2/show/NCT04542408. Accessed on 2nd June 2021.
5. Clinical Trial NCT04528888. Available from: https://clinicaltrials.gov/ct2/show/NCT04528888?term=prophylactic+anticoagulants+for+thromboembolism+in+covid-19&draw=4&rank=11. Accessed on 2nd June 2021.
6. Clinical Trial NCT04512079. Available from: https://clinicaltrials.gov/ct2/show/NCT04512079?term=prophylactic+anticoagulants+for+thromboembolism+in+covid-19&draw=4&rank=14#contacts. Accessed on 2nd June 2021.
7. Clinical Trial NCT04505774. Available from: https://clinicaltrials.gov/ct2/show/NCT04505774?term=prophylactic+anticoagulants+for+thromboembolism+in+covid-19&draw=4&rank=19#contacts. Accessed on 2nd June 2021.
8. Clinical Trial NCT04492254. Available from: https://clinicaltrials.gov/ct2/show/NCT04492254?term=prophylactic+anticoagulants+for+thromboembolism+in+covid-19&draw=4&rank=13. Accessed on 2nd June 2021.
9. Clinical Trial NCT04486508. Available from: https://clinicaltrials.gov/ct2/show/NCT04486508?term=prophylactic+anticoagulants+for+thromboembolism+in+covid-19&draw=4&rank=15. Accessed on 2nd June 2021.
10. Clinical Trial NCT04420299. Available from: https://clinicaltrials.gov/ct2/show/NCT04420299?term=prophylactic+anticoagulants+for+thromboembolism+in+covid-19&draw=2&rank=7#contacts. Accessed on 2nd June 2021.
11. Clinical Trial NCT04416048. Available from: https://clinicaltrials.gov/ct2/show/NCT04416048?term=prophylactic+anticoagulants+for+thromboembolism+in+covid-19&draw=2&rank=2. Accessed on 2nd June 2021.
12. Clinical Trial NCT04409834. Available from: https://clinicaltrials.gov/ct2/show/NCT04409834?term=prophylactic+anticoagulants+for+thromboembolism+in+covid-19&draw=4&rank=16#contacts. Accessed on 2nd June 2021.
13. Clinical Trial NCT04408235. Available from: https://clinicaltrials.gov/ct2/show/NCT04408235?term=prophylactic+anticoagulants+for+thromboembolism+in+covid-19&draw=4&rank=12#contacts. Accessed on 2nd June 2021.
14. Clinical Trial NCT04406389. Available from: https://clinicaltrials.gov/ct2/show/NCT04406389?term=prophylactic+anticoagulants+for+thromboembolism+in+covid-19&draw=5&rank=21#contacts. Accessed on 2nd June 2021.
15. Clinical Trial NCT04401293. Available from: https://clinicaltrials.gov/ct2/show/NCT04401293. Accessed on 2nd June 2021.

16. Clinical Trial NCT04367831. Available from: https://clinicaltrials.gov/ct2/show/NCT04367831?term=prophylactic+anticoagulants+for+thromboembolism+in+covid-19&draw=2&rank=1. Accessed on 2nd June 2021.
17. Clinical Trial NCT04373707. Available from: https://clinicaltrials.gov/ct2/show/NCT04373707?term=prophylactic+anticoagulants+for+thromboembolism+in+covid-19&draw=2&rank=4. Accessed on 2nd June 2021.
18. Clinical Trial NCT04360824. Available from: https://clinicaltrials.gov/ct2/show/NCT04360824?term=prophylactic+anticoagulants+for+thromboembolism+in+covid-19&draw=4&rank=18#contacts. Accessed on 2nd June 2021.
19. Clinical Trial NCT04345848. Available from: https://clinicaltrials.gov/ct2/show/NCT04345848?term=prophylactic+anticoagulants+for+thromboembolism+in+covid-19&draw=2&rank=9. Accessed on 2nd June 2021.
20. Clinical Trial NCT04662684. Available from: https://clinicaltrials.gov/ct2/show/NCT04662684?term=anticoagulant+therapy+in+patients+with+COVID-19&cond=Venous+Thromboembolism&draw=2&rank=1. Accessed on 2nd June 2021.
21. Clinical Trial NCT04508023. Available from: https://clinicaltrials.gov/ct2/show/NCT04508023?term=covid-19&cond= thrombotic +events & draw =2&rank=2. Accessed on 2nd June 2021.
